# How and when plume zonation appeared during the 132 Myr evolution of the Tristan Hotspot

**DOI:** 10.1038/ncomms8799

**Published:** 2015-07-27

**Authors:** Kaj Hoernle, Joana Rohde, Folkmar Hauff, Dieter Garbe-Schönberg, Stephan Homrighausen, Reinhard Werner, Jason P. Morgan

**Affiliations:** 1 GEOMAR Helmholtz Centre for Ocean Research Kiel, Wischhofstrasse 1-3, 24148 Kiel, Germany; 2 CAU Kiel University, Institute of Geosciences, Ludewig-Meyn-Strasse 10, D-24118 Kiel, Germany; 3 Royal Holloway, University of London, Department of Earth Sciences, Egham Hill, Egham TW20 0EX, UK

## Abstract

Increasingly, spatial geochemical zonation, present as geographically distinct, subparallel trends, is observed along hotspot tracks, such as Hawaii and the Galapagos. The origin of this zonation is currently unclear. Recently zonation was found along the last ∼70 Myr of the Tristan-Gough hotspot track. Here we present new Sr–Nd–Pb–Hf isotope data from the older parts of this hotspot track (Walvis Ridge and Rio Grande Rise) and re-evaluate published data from the Etendeka and Parana flood basalts erupted at the initiation of the hotspot track. We show that only the enriched Gough, but not the less-enriched Tristan, component is present in the earlier (70–132 Ma) history of the hotspot. Here we present a model that can explain the temporal evolution and origin of plume zonation for both the Tristan-Gough and Hawaiian hotspots, two end member types of zoned plumes, through processes taking place in the plume sources at the base of the lower mantle.

## Abstract (web summary)

Striped geochemical zonation has been observed along parts of hotspot tracks, although its origin is not well-understood. Here, the authors present Sr–Nd–Pb–Hf isotope data and present a model that can explain the evolution of zonation in both Tristan-Gough and Hawaiian hotspots, reflecting two end members.

## Graphical abstract

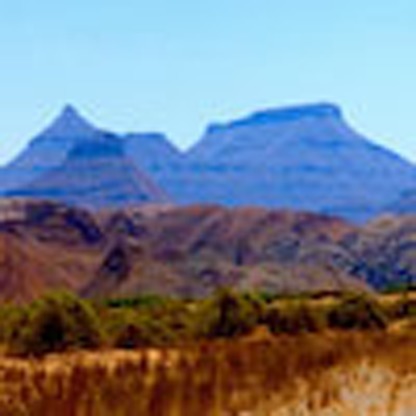

Although it has been proposed that some plumes have had constant compositions through time, such as the Louisville^1^ and Kerguelen^2^,^3^, spatial geochemical zonation has been found in a variety of hotspot tracks in the Pacific Ocean. These include the Galapagos^4^,^5^,^6^,^7^, Hawaiian^8^,^9^, Samoan^10^, Marquesan^10^,^11^ and Society^12^ hotspot tracks, which are believed to be located at the border of the large, lower mantle, low shear-wave velocity province (LLSVP) beneath the southern Pacific^9^,^10^,^12^,^13^. The longevity of plume zonation is variable for the different Pacific hotspots. It can be traced for 15–20 Myr along the Galapagos hotspot track^6^,^7^, for ∼4 Myr along the Society hotspot track^12^,^14^, for ∼5.5 Myr along the Marquesas^11^,^15^ and for up to 2 Myr along the Samoan Islands^10^,^14^,^16^. The coexistence of the geochemically distinct Loa and Kea trends of the Hawaiian mantle plume has been traced for the last 5 Myr along the island chain^9^. Little evidence exists for the presence of the Loa component in the Hawaiian seamounts, located west of the Hawaiian Islands and only the Kea component has been recognized so far in the Emperor Seamounts and older accreted Hawaiian complexes in Kamchatka^17^. To address questions concerning the longevity of plume zonation and how it originates, we investigate the entire history of the Tristan-Gough hotspot here and compare it with what is known about the history of the Hawaiian hotspot.

The Tristan-Gough hotspot track (Fig. 1) represents the classic evolution of a hotspot^18^ with active volcanic islands at its young end and flood basalt provinces at its older end. It has been termed one of the seven hotspots most likely to be derived from the lowermost mantle from a ‘primary' plume^19^ with its base currently located at the margin of the African LLSVP^20^,^21^. The emplacement of a Tristan-Gough plume head at the base of the Gondwana lithosphere caused massive volcanism forming the Parana (eastern South America) and Etendeka (western Africa) flood basalts at ∼132 Ma^22^ and may have contributed to the breakup of Africa from South America and the formation of the South Atlantic Ocean basin^23^. As the Atlantic opened, the ridge-centred plume tail formed the Walvis Ridge on the African Plate and the Rio Grande Rise (possibly underlain by a thinned continental block) on the South American Plate. As the South Atlantic mid-ocean ridge drifted westwards away from the plume tail ∼50–60 Ma, the hotspot became intraplate forming the Guyot Province, consisting of separate volcanic tracks leading to the active volcanic islands of Tristan da Cunha and Gough^24^.

Recently published Sr–Nd–Hf–Pb isotope data of samples from the Tristan-Gough hotspot track reveal that bilateral chemical zonation can be traced from the Tristan da Cunha and Gough island groups along the Tristan and Gough subtracks for the last ∼70 Myr to the southwestern (SW) end of the Walvis Ridge^13^ (Fig. 1). The enriched Gough domain exhibits higher ^207^Pb/^204^Pb for a given ^206^Pb/^204^Pb and extends to lower ^143^Nd/^144^Nd and ^176^Hf/^177^Hf ratios and generally higher ^87^Sr/^86^Sr ratios compared with the more depleted (less enriched) Tristan domain. The domains form distinct fields on the uranogenic Pb isotope diagram (Fig. 2a). Published geochemical data are, however, insufficient to establish chemical zonation beyond 70 Ma.

Here we present new Sr–Nd–Hf–Pb double spike (DS) isotope data from additional samples from the Guyot Province (obtained during R/V Akademik Kurchatov, AII-93 Atlantis II, ANT-XXIII/5 Polarstern, VM29 Vema, 51 S.A. Agulhas and SO233 Sonne cruises) and Deep Sea Drilling Programme (DSDP) Sites 525A, 527 and 528 at the SW end of the Walvis Ridge to test whether this part of the hotspot track is indeed zoned. More importantly we present new data from dredge (VM29 Vema, CIRCE Argo, AII–93 Atlantis II, CH19 Jean Charcot, WALDAA–002 Jean Charcot, RC11 Robert D. Conrad and SO233 Sonne cruises) and DSDP Leg 72 drill samples from the older part of the hotspot track (central and northeast Walvis Ridge and Rio Grande Rise) to determine whether the zonation can be traced further back in time, that is, going northeast along the hotspot track. Finally, we evaluate published data from the Etendeka and Parana flood basalts to assess the composition of the plume during its earliest history. These results are used to establish a model for the entire temporal (last 132 Myr) geochemical evolution of the Tristan-Gough hotspot. Finally, we compare the evolution of the Tristan and Hawaiian plumes and evaluate the origin of geochemical zonation in both plumes.

## Results

### Isotopic composition of Tristan-Gough hotspot track lavas

The new Sr–Nd–Hf–Pb isotope data are presented in (Table 1). We report details about the samples, the major and trace element data and isotope data with errors in Methods and Supplementary Dataset 1. The Sr–Nd–Hf–Pb isotope data of the new samples from the Guyot Province and DSDP Sites 525A, 527 and 528 at the SW end of the Walvis Ridge confirm the spatial geochemical zonation of the ≤70 Ma hotspot track proposed by (13; Fig. 2a). The data from the older (>70 Ma) hotspot track, central and northeast portion of the Walvis Ridge and the Rio Grande Rise^24^, plot exclusively within the Gough field. They also completely cover the range of the ≤70 Ma Gough track, showing that there is no systematic temporal change in the Gough source through time. The Rio Grande Rise samples and some samples from the Walvis Ridge are isotopically as enriched as DSDP Site 525A: the enriched mantle one (EMI) end member in the South Atlantic^25^,^26^.

### Sr and Nd isotopes of Etendeka and Parana flood basalts

Evaluating the composition of the mantle plume head linked to the 132 Ma Parana-Etendeka flood basalt event is difficult because of potential interaction of melts with the continental lithosphere (both crust and mantle), which overall has a geochemically enriched composition in comparison with oceanic lithosphere. Lavas and dykes associated with the 132 Ma volcanic event range from pricritic and tholeiitic basalts through highly silica-undersaturated nephelinites to highly evolved rhyolites through phonolites. Intrusive equivalents range from gabbros to granites to syenites. These magmatic rocks show an extremely large range in measured ^87^Sr/^86^Sr of ∼0.703–0.743 (with one sample having 0.924) and ^143^Nd/^144^Nd of ∼0.5117–0.5129 (Figs 3 and 4a). On plots of MgO versus ^87^Sr/^86^Sr and ^143^Nd/^144^Nd isotope ratios (Fig. 3), the most magnesium-rich samples have the lowest ^87^Sr/^86^Sr and highest ^143^Nd/^144^Nd isotope ratios and show the least variation. With decreasing MgO, the range in ^87^Sr/^86^Sr and ^143^Nd/^144^Nd increases systematically with ^87^Sr/^86^Sr extending to higher and ^143^Nd/^144^Nd to lower ratios. If only samples with MgO ⩾11 wt.% (Sr>600 p.p.m.; Supplementary Fig. 1) are considered, for example, the range in ^87^Sr/^86^Sr (0.7044–0.7091) and ^143^Nd/^144^Nd (0.51240–0.51288) is considerably reduced. The much larger range present in the more evolved compositions most likely reflects derivation through continental lithospheric melting or through assimilation of such melts by differentiated mantle melts. Extensive feldspar fractionation in many highly evolved melts reduces the Sr concentration to very low values, whereas Rb continues to be incompatible in the melts and thus its concentration increases, resulting in very high Rb/Sr ratios. Radiogenic ingrowth in the evolved rocks with very high Rb/Sr ratios contributes to the elevated ^87^Sr/^86^Sr ratios. Although the ^143^Nd/^144^Nd of the mafic (MgO ⩾11 wt.%) flood basalts is similar to the oceanic Tristan-Gough track rocks (Fig. 4a), the ^87^Sr/^86^Sr in some mafic samples extends to higher ratios, which can be explained by small amounts of assimilation of crust with very radiogenic ^87^Sr/^86^Sr (for example, some crustal rocks have extreme ^87^Sr/^86^Sr up to 1.18) and/or through melting of enriched portions of the lithospheric mantle with radiogenic Sr and Pb isotope ratios^27^.

### Pb isotopes of Etendeka and Parana flood basalts

In contrast to ^87^Sr/^86^Sr and ^143^Nd/^144^Nd where both upper and lower crustal interaction generally cause an increase in ^87^Sr/^86^Sr and decrease ^143^Nd/^144^Nd isotope ratios^27^, Pb isotope ratios do not show good correlations with MgO or Pb concentration. The reason in part for the lack of correlation is that the lower crust generally has unradiogenic Pb isotope ratios, as indicated for example by the composition of granulites from the Namaqua-Natal Belt in South Africa^28^ and diorites derived from lower crustal melting during the Damara Orogen in Namibia^29^, whereas the upper crust extends to more radiogenic Pb isotope ratios, for example, Khan granodiorites and Kuiseb schists in Namibia^30^,^31^. On the uranogenic Pb isotope diagram (Fig. 4b), where the Tristan and Gough domains form distinct fields, the Parana-Etendeka data form an array that completely covers the Gough field but extends to more radiogenic (probably reflecting upper crustal contamination) and less radiogenic (probably reflecting lower crustal contamination) compositions. Some of this variation, for example, the lower trend towards more radiogenic Pb isotope ratios (Fig. 4b), could also result from contamination within the subcontinental lithospheric mantle, which can have more radiogenic Sr and Pb but similar Nd isotope ratios^27^ to the Gough volcanic rocks. Surprisingly, no samples plot within the Tristan field. If only flood basalts that do not show clear signs of contamination in ^87^Sr/^86^Sr (defined as ^87^Sr/^86^Sr<0.7067 because 0.7067 is the highest value in the oceanic hotspot track) are considered on the uranogenic Pb isotope diagram, they only show a slightly greater range than the Gough field, which may reflect slight Pb-isotopic contamination from the continental crust. The similarity in Pb-isotopic composition between the flood basalts with low ^87^Sr/^86^Sr and the oceanic Gough domain is consistent with the initial plume material having primarily had a Gough-type composition. In summary, Parana-Etendeka samples that show no obvious signs of being contaminated within the continental lithosphere plot within the Gough domain without any evidence for the presence of Tristan-type compositions in their source.

## Discussion

Now we will evaluate possible models for explaining the origin of the Tristan and Gough domains and geochemical evolution of the Tristan-Gough hotspot. On the ^87^Sr/^86^Sr versus ^143^Nd/^144^Nd, ^143^Nd/^144^Nd versus ^176^Hf/^177^Hf, and the ^206^Pb/^204^Pb versus ^207^Pb/^204^Pb, ^87^Sr/^86^Sr, ^143^Nd/^144^Nd and ^176^Hf/^177^Hf isotope correlation diagrams (see 13, Figs 2a and 4 and Supplementary Fig. 2), the Tristan domain falls between the Gough and Atlantic MORB fields, suggesting interaction between a Gough-type plume and depleted upper mantle to generate the Tristan domain^13^. If this were the case, however, the Tristan domain would also have to be shifted towards the Atlantic MORB field on the thorogenic Pb isotope diagram (Fig. 2b), since the denominator of the plotted ratios is the same element. Instead the Tristan domain almost completely overlaps the Gough domain and both are distinct from Atlantic MORB, in stark contrast to the nearly complete separation of the domains on the uranogenic Pb isotope diagram on which the Tristan domain overlaps the Atlantic MORB field (Fig. 2a). Therefore, despite being generally more geochemically depleted in composition than the Gough domain, the Tristan domain cannot simply be derived through mixing of a Gough source and upper MORB-source mantle. Consequentially, we attribute both compositional types to the Tristan–Gough plume, implying a deep source for both.

To evaluate the origin of plume zonation, a fundamental question concerns the distribution of both compositional types in the Tristan–Gough plume. Is the plume chemically zoned with distinct Tristan and Gough domains^13^ or are these components uniformly distributed throughout the plume but sampled by different extents of melting, for example, under lithosphere with different thicknesses^32^,^33^,^34^? In particular, was the Tristan composition also present in the early plume (∼70–132 Ma), but simply not sampled? Picritic and tholeiitic flood basalts, generated during the initial plume-head stage of volcanism, are typically associated with high temperatures and thus high degrees of melting (commonly 20–30%)^35^ even in continental settings^35^,^36^. Therefore, the Etendeka and Parana flood basalts should have preferentially sampled the more depleted (or less enriched) Tristan component, if it were present. Even if the Tristan component was somehow more fertile (despite being more depleted isotopically), the lower-degree alkalic melts (alkali basalts, basanites and nephelinites) associated with the flood basalt volcanism should have sampled the more fertile component, if both components had been present. Since so far only Gough compositions have been identified in the flood basalts, despite the large range in rock types reflecting a large range of melting conditions, the plume head is likely to have primarily had a Gough-type composition.

Although temperatures may have been lower during the formation of the early hotspot track from the plume stem than during the plume-head stage, the early-plume stem was located beneath the mid-Atlantic ridge, allowing upwelling to shallower depths and greater extents of pressure-release melting than possible beneath continental lithosphere. This situation should also have favoured melting of the more depleted Tristan component if it had been present in the plume. Certainly compared with the later, intraplate history of the hotspot (<60 Ma), degrees of melting should have been higher during melting of the early plume stem. Ratios of more to less incompatible trace elements (for example, La/Sm, which is relatively insensitive to differentiation except in highly evolved rocks) are inversely proportional to the melt fraction. Therefore, if lithospheric thickness controls the extent of melting, the La/Sm ratio should have been lower when the plume was ridge-centred and should have increased as the plume became progressively more intraplate after ∼60 Ma. As expected, the La/Sm ratio shows a very crude overall increase with decreasing age along the hotspot track (Supplementary Fig. 3), consistent with higher degrees of melting while the plume was ridge-centred. As noted previously, higher degrees of melting should have favoured melting of the more depleted Tristan component if it had been present in the plume during its earlier (∼70–120 Ma) history. Finally, parental magma types range from tholeiite to alkali basalt on the Walvis Ridge and Rio Grande Rise. Therefore, if both Tristan and Gough components were present in the early plume stem (∼70–120 Ma), it is likely that both would have been sampled regardless of which was more fertile^37^,^38^. Since none of the samples older than 70 Ma have clear Tristan-type compositions, we do not believe that the Tristan component, if present at all, was abundant in the early plume.

**Figure 1:**
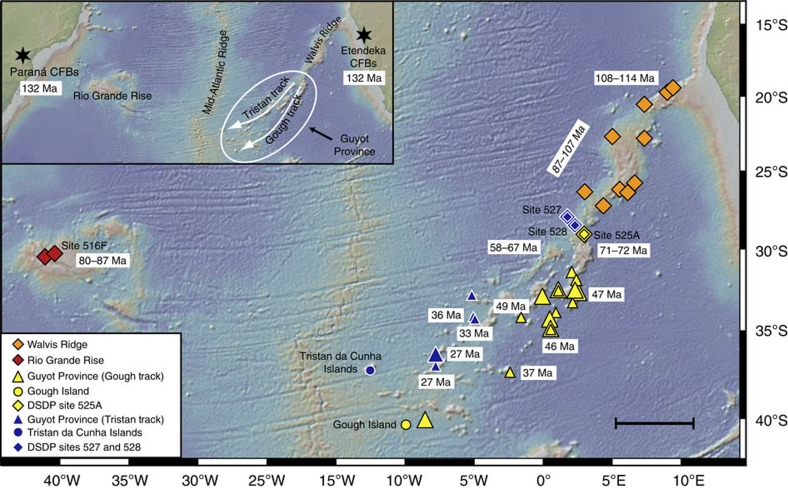
Bathymetric map of the South Atlantic Ocean and its margins showing the Tristan-Gough hotspot track. The map shows the Tristan-Gough hotspot track including the ∼132 Myr (ref. 22) old Etendeka and Parana continental flood basalt provinces, the Rio Grand Rise and Walvis Ridge (both within the age range ∼60–115 Ma), the Guyot Province at the southwestern end of the Walvis Ridge containing the Tristan da Cunha and Gough Island groups (∼0–60 Ma)^24^. Ages for late-stage volcanism are not shown. Age range in italics (87–107 Ma) is estimated using a spatial age progression equation^24^. Sample locations are denoted by symbols: large symbols this study and small symbols as reported in ref. 13. Source of base map is http://www.geomapapp.org. All sites are from the Deep Sea Drilling Project. The scale bar in the lower right hand corner indicates a distance of ∼500 km.

**Figure 2:**
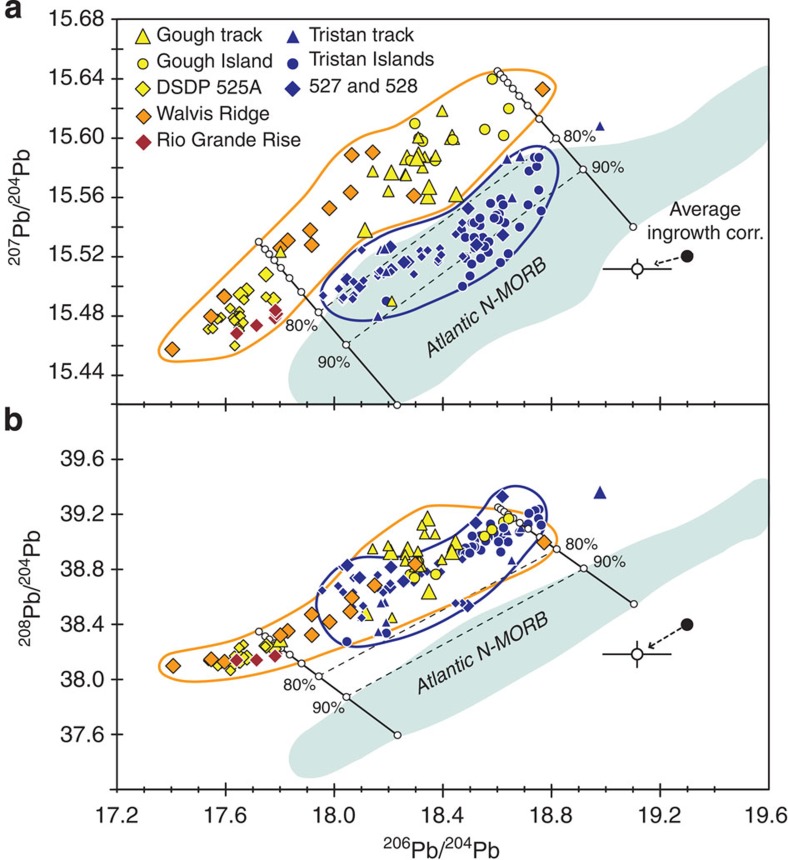
The >70 Ma Tristan-Gough hotspot track lavas have Gough-type isotopic compositions. On the (**a**) ^206^Pb/^204^Pb versus ^207^Pb/^204^Pb (uranogenic Pb) isotope diagram, older hotspot lavas (>70 Ma) fall solely within the Gough field of ref. 13. Even though they are completely separated on the uranogenic Pb isotope diagram, the Tristan and Gough fields completely overlap on the (**b**) ^206^Pb/^204^Pb versus ^208^Pb/^204^Pb (thorogenic Pb) isotope diagram, indicating that the Tristan compositions cannot simply be explained by mixing of Gough compositions with Atlantic MORB. Modelling shows that addition of 70 to >90% Atlantic N-MORB to Gough can explain the shift in Tristan to N-MORB in (**a**), but would require the Tristan field to be shifted towards Atlantic N-MORB in (**b**), for example, extending beyond the >90% mixing line towards N-MORB as in (**a**). Pb concentrations used for the mixing calculations are 0.57 p.p.m. for the MORB end members (based on average of global N-MORB^62^) and 3.2 p.p.m. for the Gough end members (average of Gough samples with MgO>1 wt.%) (Supplementary Dataset 1). Changing the assumed concentrations for Gough and MORB end members will simply shift the mixing percentages, but mixing of Gough and Atlantic N-MORB will still require Tristan to be shifted towards N-MORB compared with Gough on the thorogenic Pb isotope diagram. To compare isotope data of samples ranging in age between ∼0–115 Ma, we use the measured compositions, assuming that the parent-daughter ratios were not significantly fractionated during melting or subsequent differentiation. The ‘average ingrowth corr.' and associated 1*σ* variation is the average correction needed for radiogenic ingrowth for the plotted samples (Supplementary Dataset 1). Since the age correction moves the data sub parallel to the boundary between the Gough and Tristan fields in (**a**), radiogenic ingrowth does not cause overlap between Gough and Tristan samples and therefore does not affect the classification of the samples. Analytical errors of data (Supplementary Dataset 1) in these and all subsequent isotope diagrams are smaller than symbol size. Numbers in the legend refer to DSDP Sites (Fig. 1). Atlantic MORB from PetDB (http://www.earthchem.org/petdb) and additional Tristan-Gough data from GEOROC (http://georoc.mpch-mainz.gwdg.de/georoc/).

**Figure 3:**
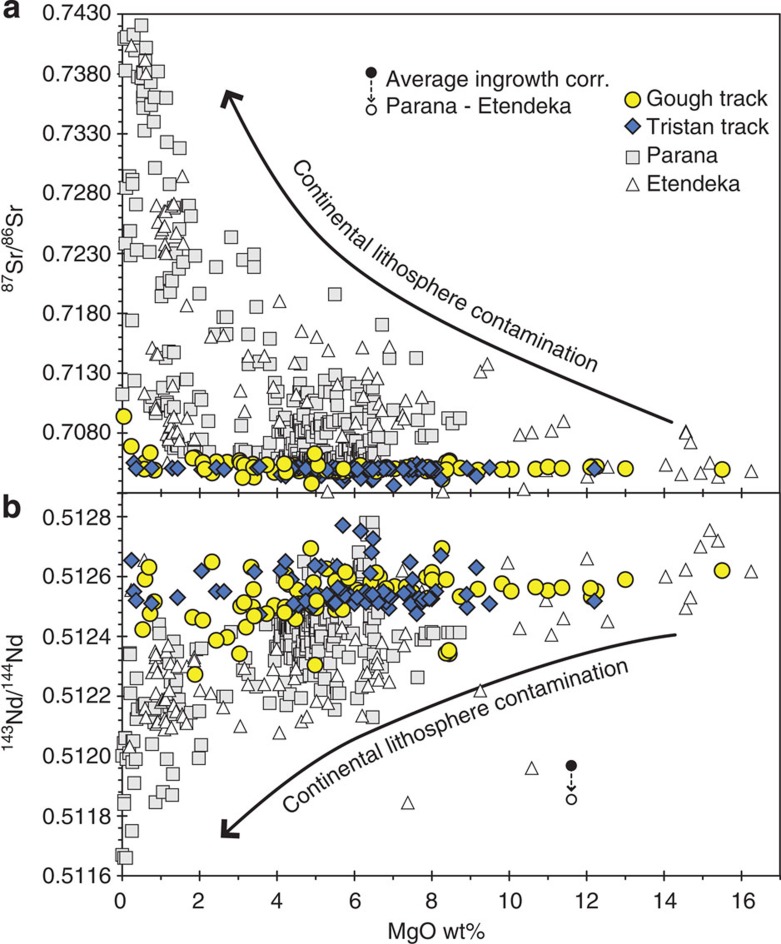
Evolved Parana and Etendeka continental flood volcanism shows much greater isotopic variation than the oceanic Tristan-Gough hotspot track lavas. On plots of MgO vs. (**a**) ^87^Sr/^86^Sr and (**b**) ^143^Nd/^144^Nd, the oceanic lavas have relatively constant composition and unradiogenic ^87^Sr/^86^Sr but radiogenic ^143^Nd/^144^Nd regardless of MgO content (degree of differentiation). The continental volcanism, however, shows a greater range in ^87^Sr/^86^Sr and ^143^Nd/^144^Nd extending to systematically more radiogenic ^87^Sr/^86^Sr and less radiogenic ^143^Nd/^144^Nd with increasing degree of differentiation (decreasing MgO). The increasing and substantially greater range in ^87^Sr/^86^Sr and ^143^Nd/^144^Nd for the continental, compared with oceanic, volcanic rocks primarily reflects increasing amounts of assimilation during fractional crystallization of continental lithosphere by some magmas. Radiogenic ingrowth in some of the most evolved silica-saturated samples with very high Rb/Sr ratios also contributes to the extremely radiogenic ^87^Sr/^86^Sr. Average correction for radiogenic ingrowth for Parana and Etendeka is based on 498 (^87^Sr/^86^Sr) and 280 (^143^Nd/^144^Nd) analyses for which parent/daughter ratios are available. See Supplementary Dataset 5 and additional data from GEOROC (http://georoc.mpch-mainz.gwdg.de/georoc/).

**Figure 4:**
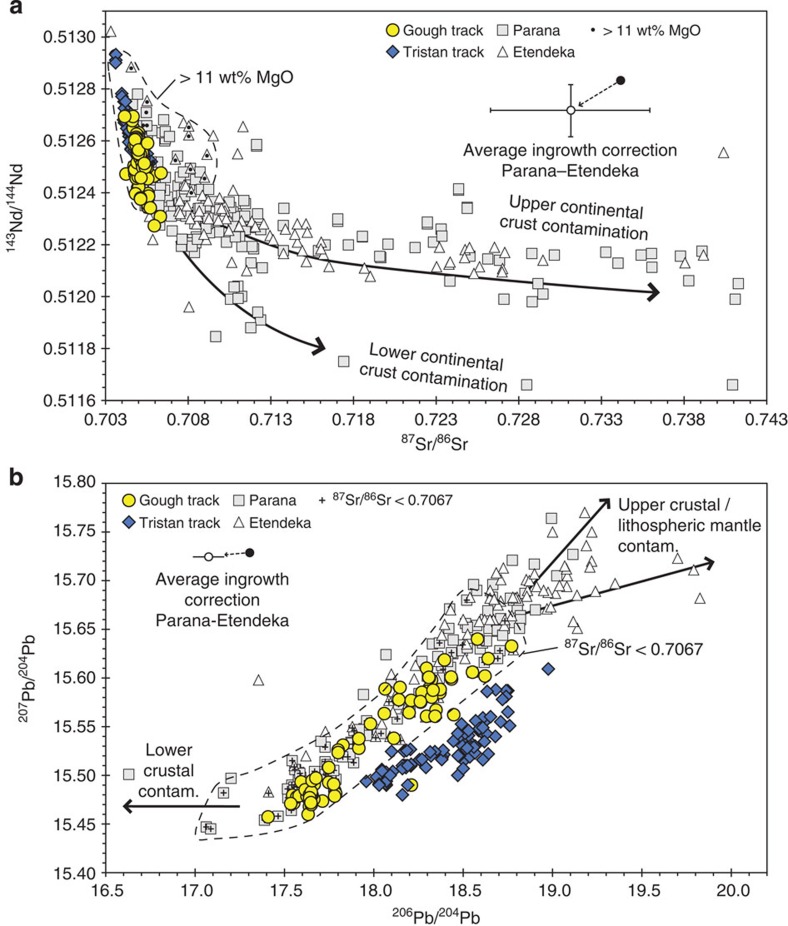
Larger range in isotopic composition of the continental flood volcanism most likely reflects continental lithospheric contamination. The range in isotopic composition of continental flood volcanism and Tristan-Gough oceanic hotspot track volcanism are shown on (**a**) ^87^Sr/^86^Sr versus ^143^Nd/^144^Nd and (**b**) ^206^Pb/^204^Pb versus ^207^Pb/^204^Pb isotope correlation diagrams. In (**a**) samples with MgO>11 wt.% (enclosed within dashed line) show a much more restricted range in isotopic composition, yet ^87^Sr/^86^Sr ratio in some mafic samples is still higher than in the oceanic part of the hotspot track, either reflecting contamination by crustal material or lithospheric mantle, both of which can have extremely radiogenic Sr^27^,^28^. In (**b**), if only Etendeka and Parana flood basalts with ^87^Sr/^86^Sr<0.7067 (highest value in the oceanic hotspot track) are considered on the uranogenic Pb isotope diagram (marked with a cross and enclosed within the field defined by the dashed line), they only show a slightly greater range than the Gough field, suggesting that ^87^Sr/^86^Sr ratio can be used to effectively filter for continental lithospheric contamination. Arrows denote directions for upper and lower crustal and/or lithospheric mantle contamination. In (**a**) the arrow labelled ‘Upper continental crust contamination' extends into the field for Damara S-type granites and points to the field for Damara metasediments^27^. The arrow labelled ‘Lower continental crust contamination' points toward the Kaokoland gneisses (Pre-Damara basement)^27^. In (**b**) the ‘Lower crustal contam. (contamination)' arrow overlaps with and points to lower crustal granulites from the Namaqua-Natal Belt in South Africa^28^. The ‘Upper crustal/lithospheric mantle contamination' arrows point to the following rock groups in Namibia: (1) upper arrow–Khan granodiorites (samples G12 and G13 (ref. 30) and 02/99 and 03/99 (ref. 31)) and (2) lower arrow–Kuiseb schists (sample Kh27 (ref. 30)) and lithospheric mantle, estimated to have a present-day composition of ^206^Pb/^204^Pb∼19.8 and ^207^Pb/^204^Pb ∼15.7 (based on sample VB32) beneath the Spitzkoppe region in Namibia^27^. See Supplementary Dataset 5 and additional data from GEOROC (http://georoc.mpch-mainz.gwdg.de/georoc/). Average radiogenic ingrowth correction and 1*σ* variation for Parana and Etendeka as defined in the Fig. 3 caption.

Over the last ∼70 Myr, the hotspot track consisted of distinct Tristan and Gough compositional geographic domains with the northwestern portion of the hotspot track (going to Tristan da Cunha Island) consisting of Tristan-type compositions and the southeastern part of the track (going to Gough Island) consisting of Gough-type compositions^13^ (Fig. 1). The first known occurrence of Tristan-type lavas occurred near the SW end of the Walvis ridge when the hotspot was still ridge centred. After ∼60 Ma, the plume became intraplate and the lithosphere overlying the hotspot generally became thicker with decreasing age^13^. Since the temporal variations in lithospheric thickness over the last 70 Myr far outweigh the spatial differences in the lithospheric thickness between the Tristan and Gough subtracks at any given time, we would expect a temporal rather than a spatial change in isotopic composition. We, however, do not see a shift in isotopic composition from one type of component to the other with age, but rather see a spatial separation throughout the last 70 Myr of the hotspot's history. Although minor compared with the changes in lithospheric thickness with age, the Tristan track was located slightly closer to the Mid-Atlantic ridge during its formation and thus on slightly younger and thinner crust than the Gough track, which suggests that it may have formed through higher degrees of melting. If this were the case, we would expect more to less incompatible element ratios to be systematically lower in the Tristan lavas than in the Gough lavas. The La/Sm ratios of the lavas from the two subtracks, however, largely overlap at any given time or position along the hotspot track (Supplementary Fig. 3), suggesting that potential differences in lithospheric thickness between the two domains did not lead to a systematic difference in degree of melting. This is not surprising since the fractionated and overlapping heavy rare earth element patterns in both the Tristan and Gough lavas^13^ suggest that garnet was a residual phase during melting for both compositional types, and thus melting was likely to be well below the depths affected by the thickness of young lithosphere. In summary, since the isotopic composition of the erupted lavas does not appear to be related to differences in melting conditions and extent, we conclude that the plume primarily contained a Gough-type compositional range during its early history with a Tristan vertical compositional zone or stripe^39^,^40^ appearing on its northwestern side ∼70 Ma (ref. 13). Thereafter the plume remained bilaterally zoned. Below we discuss models to explain how a plume with relatively uniform composition turned into a zoned plume half way through its lifetime.

Although previous models have favoured the base of the lower mantle to be the source of plume zonation^6^,^8^,^9^,^10^,^12^,^13^, we will first consider mid-mantle origins for the compositional variation. Plumes, for example, could entrain a sheath of depleted MORB-like material when they pass through the upper–lower mantle transition zone^41^,^42^. Thus, we must pose the question as to whether the Tristan component could have been picked up in the transition zone. In material with a strongly temperature-dependent viscosity like the earth's mantle, however, laboratory and numerical experiments suggest much smaller amounts of entrainment than for thermal plumes in an isoviscous fluid^40^. Seismic imaging of examples of apparent plume broadening beneath 660 km suggests that there may be a higher viscosity transition zone^43^. This type of radial mantle viscosity structure, however, was also found to be associated with limited entrainment^40^. Furthermore, the high ^208^Pb/^204^Pb of the Tristan component distinguishes it from depleted upper mantle and it is hard to explain why an entrained sheath of material around the entire plume would only be sampled by melting on the plume's NW side, in particular when the plume was intraplate. Perhaps stalled slabs could be distributed irregularly in the transition zone, so that the plume may only have entrained such material on its NW side^44^. Recycled ocean crust is commonly believed to have a high time-integrated U/Pb ratio generating radiogenic ^206^Pb/^204^Pb at relatively unradiogenic ^208^Pb/^204^Pb over time and thus plotting beneath an extension of the MORB field^26^. The Gough and Tristan components, however, have relatively low ^206^Pb/^204^Pb and high ^208^Pb/^204^Pb and plot above the MORB field on the thorogenic Pb isotope diagram. In conclusion, mid-mantle entrainment models do not appear to be able to adequately explain the zonation of the Tristan-Gough plume.

**Figure 5:**
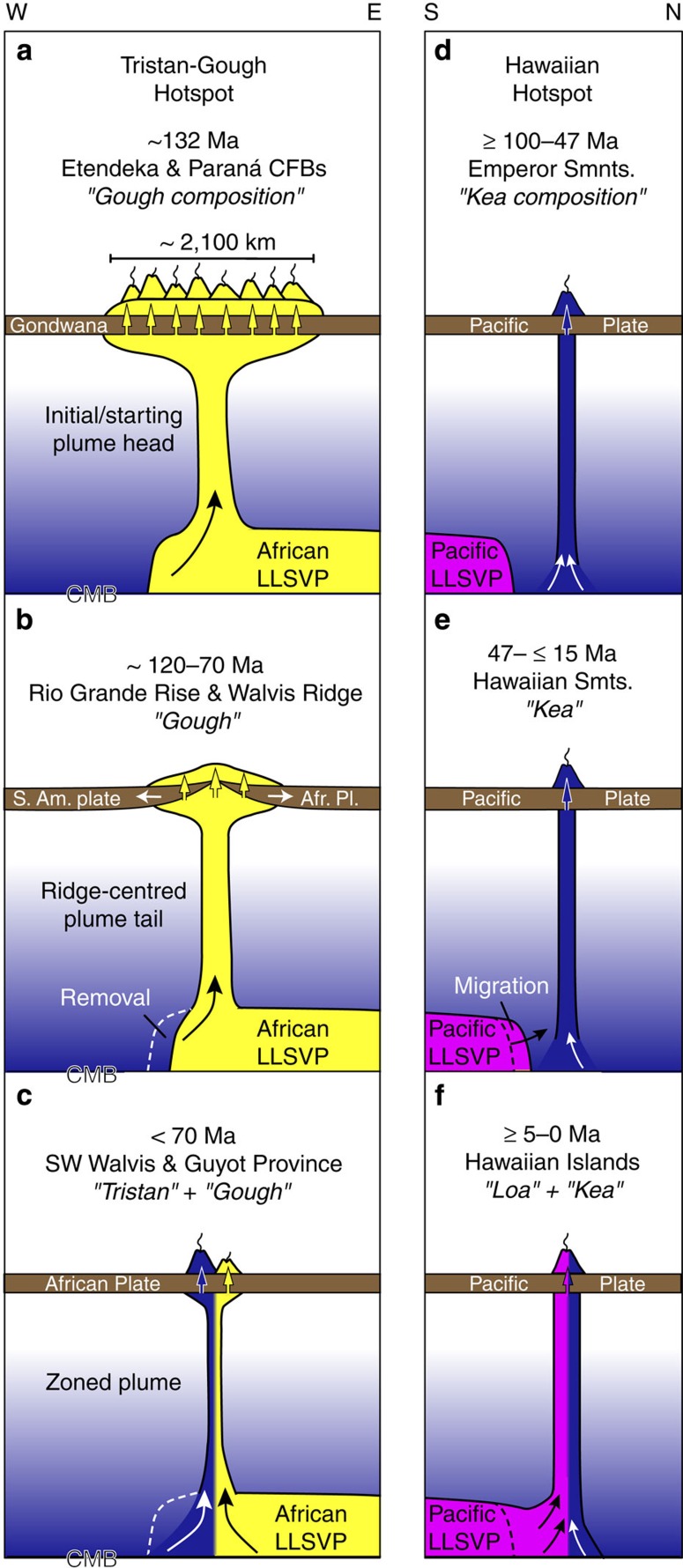
Models illustrating the geochemical evolution of the Tristan-Gough and Hawaiian hotspots. (**a**) For the Tristan-Gough hotspot, the plume head is derived from the inner margin of the African low shear-wave velocity province (LLSVP) with enriched Gough composition. (**b**) The early plume stem continues to tap only LLSVP, but the margin of the LLSVP is continually drawn closer to the plume stem and/or the plume stem migrates towards the LLSVP boundary. (**c**) At ∼70 Ma, the LLSVP material on the northeastern side of the plume conduit is exhausted or the base of the plume may have migrated to the LLSVP boundary, so that depleted Tristan material from outside the LLSVP is also drawn into the plume conduit resulting in a zoned plume. (**d**) There is no geological evidence for the initiation of the Hawaiian plume. The oldest volcanic rocks associated with the hotspot are from ∼100 Myr old seamounts accreted to the forearc in Kamchatka^17^. The Hawaiian plume originally only taps the ambient depleted lower mantle (Kea component) between ∼100–47 Ma. (**e**) At some time between 45 and 5 Ma (most likely between ⩾15 and 7 Ma when there was a peak in volcanic flux from the Hawaiian plume), enriched Loa material is drawn into the Hawaiian plume from the Pacific LLSVP (which has a distinct composition from the African LLSVP) and the plume becomes zoned. (**f**) After ⩾15–5 Ma the Hawaiian Plume has remained zoned. S. Am., South American; Afr. Pl., African plate; CMB, core-mantle boundary. Profiles are not to scale. No distinction has been made between the thickness of oceanic and continental lithosphere.

We propose the following model to explain the observed temporal and spatial changes in geochemistry of the Tristan-Gough hotspot track (Fig. 5). Both analogue^40^ and numerical^39^,^40^,^45^ experiments suggest that thermal plumes and starting plumes in the mantle will primarily consist of material from their basal boundary layer. According to plate motion reconstructions, the original eruption site of the Parana–Etendeka flood basalts lies vertically above and well within (∼1,000 km) the western edge of the African LLSVP, defined by the 1% slow shear-wave velocity contour^20^,^21^. The Tristan–Gough plume head is therefore likely to have been derived from the African LLSVP and, as dynamic plumes are near-vertical flow structures, its base will have sampled this region, consistent with this part of the LLSVP having a Gough-type source composition. In contrast, in these reconstructions the present Tristan-Gough hotspot is located south-southwest of the reconstructed flood basalt source region so that it now almost directly overlies the boundary of the African LLSVP. If these reconstructions are correct, the change in location could reflect migration of the plume stem towards the margin of the LLSVP after the initial plume head event at an average relative speed of ∼1,000 km per 100 Myr or ∼1 cm per year. This scenario implies that both plume stem locations and the boundary of the LLVSP can slowly drift due to slow deep mantle flow^46^ moving at speeds of ∼1–10 mm per year, <10% of typical speeds of surface plate motions. Alternatively, the base of the plume may have remained fixed in location throughout its early history. In this case, the plume may have exhausted the Gough material between the plume base and the margin of the LLSVP at ∼70 Ma, sucking the LLSVP boundary into the base of the plume. A similar model has been proposed to explain the appearance of the Loa stripe in the Hawaiian plume, in which the Hawaiian plume draws in mantle from the Pacific LLSVP (Fig. 5)^47^. The appearance of Tristan-type compositions at ∼70 Ma in the Tristan-Gough hotspot track suggest that the boundary of the Atlantic LLSVP was drawn into the base of the plume at a northeastern (NE)-SW orientation, resulting in a bilaterally zoned plume assuming laminar flow in the plume conduit^6^,^39^,^40^,^45^,^47^,^48^. For largely NE-SW absolute plate motions, surface geochemical zonation is predicted by numerical experiments to form in a NE-SW direction, reflecting the inflow pattern at the base of the plume stem^47^. Since plume material is thought to rise within the stem at speeds >1 m per year (refs 46, 49), it would have taken <3 Myr for Tristan-type mantle to appear in the hotspot melts after this component entered the plume stem. Over the last ∼70 Myr, the distance between the Tristan and Gough subtracks has progressively increased, which could reflect progressive bifurcation of a plume that roughly split along its compositional boundary^24^. In addition, the Tristan subtrack has become a progressively more prominent volcanic feature (in respect to erupted volume) than the Gough subtrack, suggesting that the intake of Gough-type material in the Tristan-Gough plume has systematically diminished at the expense of the Tristan-type material with decreasing age. If this trend continues, Gough material could also be exhausted from the southeastern side of the plume source in the future, such that the plume becomes entirely Tristan-like in composition. This model demonstrates how plume zonation can develop and also disappear, accompanied by an overall change in the composition of the plume from before to after its zoned history.

In contrast to this postulated evolution for the Tristan-Gough hotspot track, the Hawaiian hotspot track is not associated with a known large igneous province or flood basalt event at its initiation, and we only have the geologic record of a long-lived plume tail. The Loa component, presumably derived from the Pacific LLSVP^9^,^10^, is absent in the Emperor Seamounts and older accreted Hawaiian complexes in Kamchatka, with only the Kea component being present in the earlier history of the hotspot^17^. Thus far, the Loa component has primarily been found in the Hawaiian Islands^8^,^9^,^10^,^17^, but sufficient data are not available to evaluate its presence in the Hawaiian Seamounts. If the Hawaiian plume was located near to but outside of the Pacific LLSVP, it could have sucked in LLSVP material with time^47^. This is a potential explanation for the significant increase in Hawaiian plume output (crustal volume flux) observed in the bathymetry and residual gravity anomalies beginning at ⩾15 Ma with a further marked increase at ∼7 Ma (ref. 50), although passage of fracture zones bounding lithosphere of different ages/thicknesses over the hotspot no doubt also contributed to changes in volcanic flux^51^. In conclusion, while plumes appear to form near the boundaries of the LLSVPs^20^,^21^, plume stems may form or migrate to either side of LLSVP boundaries or, alternatively, remain stationary and pull in a migrating boundary of a LLSVP. Both situations would lead to a compositionally zoned plume. Tapping of LLSVP material, if it is hotter and/or more fertile than surrounding ambient deep mantle, would lead to an increase in productivity of the plume, whereas tapping of more depleted and/or colder ambient mantle at the margin of a LLSVP would lead to a decrease in plume magma productivity. This scenario lends further support to the paradigm that plumes are long-lived upwelling structures within the convecting mantle, flow features that can persist despite a change in their source material. If so, there is the potential to further use geochemical evolution along other long-lived plume tracks to map the space-time variation of mantle composition near the core-mantle boundary.

## Methods

### Overview

Sixty dredge and drill core samples were analysed for major and trace elements and forty-one of them for Sr–Nd–Hf–Pb DS isotope ratios in the framework of this study. A full table with all analytical data and sample information is provided in Supplementary Dataset 1.

### Sampling

The samples were obtained from several repositories and were originally recovered during the US American cruises RC11 and RC16 (R/V Robert D. Conrad) in 1967 and 1972, the CIRCE cruise (R/V Argo) in 1968, cruise VM 29 (R/V Vema) in 1972, cruise AII–93 (R/V Atlantis II) in 1975, during the French cruise (WALDA–002) CH19 (R/V Jean Charcot) in 1971, during a Russian cruise with the R/V Akademik Kurchatov in 1975, during cruise 51 of the South African ship R/V S.A.Agulhas, during DSDP Legs 72 (Site 516F), 74 (Sites 525A, 527 and 528) with the R/V Glomar Challenger in 1980 and during German expeditions with the R/V Polarstern (ANT-XXIII–5 (PS69)) in 2006 and the R/V Sonne (SO233) in 2014. Forty samples are from sites located along the Walvis Ridge, including dredge samples from the northern flank at its northeastern-most end close to the Namibian coast and from the southern flank of the southwestern part, from DSDP Sites 525A, 527 and 528, which form a transect across the southwestern end of the Walvis Ridge. Nine samples were obtained from the Rio Grande Rise on which isotope analyses were carried out on five. These include two dredge sites at the border of a northwest-southeast trending canyon that cuts the Western Rio Grande Rise and from DSDP Site 516F on the main platform of the Rio Grande Rise. We also analysed ten additional primarily evolved dredge samples from the Gough subtrack and one from the Tristan subtrack within the Guyot Province.

### Sample preparation

For the geochemical analyses only the least altered inner parts of the selected rock samples were used to prepare rock chips and powders. After initial jaw-crushing, the rock chips were sieved (fractions: <0.25, 0.25–0.5, 0.5–1, 1–2, 2–4 and >4 mm) and thereafter repeatedly cleaned in an ultrasonic bath with deionized water for ∼20–30 min until a clear solution was obtained. The freshest chips were then hand-picked under a binocular microscope. About 5–10 g of 1- to 4-mm-sized whole-rock chips were taken to prepare bulk rock powders (in an agate mortar and agate ball-mill) for major and trace element and Sr–Hf isotope analyses. In addition, 500 mg of whole-rock chips (0.5–1 mm fraction) was picked under a binocular microscope for Sr–Nd–Pb isotope analyses.

### Major element analyses

Major elements were determined by X-Ray Fluorescence Analysis (XRF) at GEOMAR Helmholtz Centre for Ocean Research Kiel (using a Philips X'Unique PW 1480 X-ray florescence spectrometer), at the Institute of Mineralogy and Petrography at the University of Hamburg (using a Magix Pro PW 2540 XRF) and by inductively coupled plasma emission spectroscopy in the Acme Analytical Laboratories in Vancouver. Eleven international rock standards were measured along with the samples (JA–2, JA–3, JB–2, JB–3, JG–2, JG–3, JGB–1, JR–1, AGV–2, BIR–1, BHVO–2 and BCR–2). Results and information about data quality are presented in Supplementary Dataset 2.

### Trace element analyses

Trace element analyses were carried out on an AGILENT 7500cs inductively coupled plasma mass spectrometer at the Institute of Geosciences at the Christian-Albrechts-University of Kiel after the methods of ref. 52 and the Acme Analytical Laboratories in Vancouver by inductively coupled plasma mass spectrometry (ICP-MS) subsequent to a lithium metaborate/tetraborate fusion and nitric acid dissolution as well as an aqua regia digestion. BIR–1, BHVO–2, BCR–2 and AGV–2 were analysed as geochemical reference materials. Results and information about data quality are given in Supplementary Data set 3 (University Kiel) and Supplementary Data set 4 (Acme).

### Isotope analyses

Sr–Nd–Pb–Hf isotope analyses were carried out at GEOMAR Helmholtz Centre for Ocean Research Kiel by thermal ionization mass spectrometry (Sr–Nd–Pb) and multi-collector ICP-MS (Hf) in static multi-collector mode on both types of instruments. Between 100 and 250 mg of sample chips were leached in 2 N HCl at 70 °C for 1 h and subsequently triple rinsed in 18 MΩ water before digestion. For each sample additional Sr isotope analyses were generated on 100 mg of leached powders (6 N HCl at 150 °C for 3 days) to remove possible seawater alteration effects more thoroughly. Ion chromatography followed established standard procedures^53^,^54^. Pb and most Sr isotope analyses were performed on a Finnigan MAT 262 RPQ^2+^ while Nd and the remaining Sr isotope analyses were determined on a Thermo Fisher TRITON thermal ionization mass spectrometer. Nd and Sr ratios were normalized within run to ^146^Nd/^144^Nd=0.7219 and ^86^Sr/^88^Sr=0.1194, respectively. Total chemistry blanks were below 150 p.g. for both elements and thus negligible. Sample data are reported relative to ^87^Sr/^86^Sr=0.710250±0.000013 (*n*=42; 2*σ* external reproducibility) for NBS987 on the MAT262 RPQ^2+^ and ^87^Sr/^86^Sr=0.710250±0.000013 (*n*=5; 2*σ* external reproducibility) on the TRITON. Nd sample data are reported relative to ^143^Nd/^144^Nd=0.511850±0.000007 (*n*=25; 2*σ* external reproducibility) for La Jolla and our in-house SPEX Nd monitor ^143^Nd/^144^Nd=0.511715±0.000005 (*n*=13; 2*σ* external reproducibility). Pb mass bias correction followed the DS technique^55^. DS-corrected values for NBS981 are ^206^Pb/^204^Pb=16.9416±0.0024, ^207^Pb/^204^Pb=15.4992±0.0024 and ^208^Pb/^204^Pb=36.7246±0.0061 (*n*=18; 2*σ* external reproducibility) and compare well with published double- and triple-spike data^56^,^57^,^58^,^59^,^60^. Total chemistry blanks were 10–40 p.g. for Pb and thus negligible. Hf chemistry followed the two-column procedure^61^ using unleached powders, and analyses were carried out on a VG Axiom MC-ICPMS and on a Nu plasma MC-ICPMS. Within-run mass bias correction used for ^179^Hf/^177^Hf was 0.7325. Total chemistry blanks were between 60 and 150 p.g. Hf. Our in-house SPEX Hf ICP standard solution (Lot no.9) was calibrated to JMC 475 (^176^Hf/^177^Hf=0.282163 (ref. 61)) and gave an average standard bracketing normalized ratio of ^176^Hf/^177^Hf=0.282173±0.000008 (*n*=132; 2*σ* external reproducibility; VG Axiom MC-ICPMS) and 0.282170±0.000004 (*n*=48; 2*σ* external reproducibility; Nu plasma MC-ICPMS). Furthermore, USGS reference material BHVO–2 gave ^176^Hf/^177^Hf=0.283107, which compares well with the mean of compiled values at GEOREM (^176^Hf/^177^Hf=0.283109±0.000012; http://georem.mpch-mainz.gwdg.de/).

### Evaluation of isotope replicate analyses

Replicate analyses for Sr–Nd–Pb isotopes were carried out on a second sample digestion for five samples. Whole-rock chips were leached with warm 2 N HCl and powders with hot 6 N HCl as described above. While the reproducibility of ^143^Nd/^144^Nd lies within (three samples) or very close (two samples) to the external 2*σ* errors of the standards, offsets outside the external 2*σ* array of the standards are observed for radiogenic Sr and Pb isotope ratios on leached chips. In detail DSDP Leg 74 528 42 1 W 29–45 lies slightly outside the external errors while a larger offset is observed for PS69/424–1–DR 26–1. The somewhat limited reproducibility for Sr on leached whole-rock chips is ascribed to variable degrees of seawater alteration even in visually homogeneous-sample chips and the inability of the leaching reagent to fully penetrate the sample and thus remove all Sr introduced through secondary processes. Notably, strong leaching of powders does not always yield the least radiogenic ^87^Sr/^86^Sr when compared with the corresponding data of the leached chips. Therefore, the least radiogenic ^87^Sr/^86^Sr of each sample is plotted and displayed in Table 1 of the manuscript, since it is presumably closest to the pristine magmatic value. Pb isotope ratios are reproduced within the external 2*σ* errors for NBS981 for four out of five samples in ^207^Pb/^204^Pb and three samples in ^208^Pb/^204^Pb, while ^206^Pb/^204^Pb is reproduced for only one sample under these specifications. Interaction with seawater during low temperature alteration and seafloor weathering can lead to a heterogeneous enrichment of uranium that causes variable degrees of ^206^Pb ingrowth over time scales relevant in this paper. In this respect it is worth noting that ^207^Pb/^204^Pb is less affected by secondary U enrichment as ^235^U is 137.88 times less abundant than ^238^U presently. The two samples for which ^208^Pb/^204^Pb did not reproduce within the external 2*σ* error of NBS981 appear to have undergone a more complex alteration history that includes Pb removal at high temperatures in addition to U enrichment at low temperatures. The slight variations caused by alteration, however, do not affect the overall scientific interpretations and conclusions derived from the Sr and Pb isotope data. Replicate Hf analyses on sample DSDP Leg 74 525A 57 5 W 141–148 agreed within the external 2*σ* errors of the standard.

## Additional information

**How to cite this article:** Hoernle, K. *et al*. How and when plume zonation appeared during the 132 Myr evolution of the Tristan Hotspot. *Nat. Commun.* 6:7799 doi: 10.1038/ncomms8799 (2015).

## Supplementary Material

Supplementary Information Supplementary Figures 1-3

Supplementary Dataset 1 Correlated major element, trace element and isotope data.

Supplementary Dataset 2 Major element data of reference material.

Supplementary Dataset 3 Quality measures of trace element data by ICPMS (Institute of Geosciences Kiel University).

Supplementary Dataset 4 Trace element data of reference material by ICPMS (Acme Analytical Laboratories Ltd.).

Supplementary Dataset 5 Data plotted for the Etendeka and Parana flood basalts from GEOROC (http://georoc.mpch-mainz.gwdg.de/georoc/).

## Figures and Tables

**Table 1:** Sr–Nd–Hf–Pb double-spike isotope data for Tristan-Gough hotspot track samples.

**Sample**	**Age**	**Lat.**	**Long.**	^**87**^**Sr/**^**86**^**Sr**	^**143**^**Nd/**^**144**^**Nd**	^**206**^**Pb/**^**204**^**Pb**	^**207**^**Pb/**^**204**^**Pb**	^**208**^**Pb/**^**204**^**Pb**	^**176**^**Hf/**^**177**^**Hf**
***Tristan Track***									
AK–1695–6	**27**	−36.45	−7.81	—	0.512654	18.9762	15.6093	39.3651	0.282835
									
***Gough track***									
SO233 DR3–1	*66*	−32.86	2.50	0.705358	0.512483	18.2685	15.5852	38.9360	—
AII–93–11–8	*47*	−32.97	−0.02	—	0.512631	18.1120	15.5381	38.4782	0.282799
PS69/420–1–DR 21–1	**47**	−32.81	2.55	—	0.512423	18.3429	15.5607	39.1694	0.282682
PS69/423–1–DR 25–4	**46**	−34.92	0.55	0.706357	0.512476	18.2090	15.5770	38.9231	0.282704
PS69/424–1–DR 26–1	*46.5*	−34.92	0.55	0.704158	0.512694	18.4471	15.5623	38.9972	0.282841
V29–9–1	*47*	−32.63	1.12	0.704664	0.512694	18.3477	15.5671	38.6352	0.282822
AG51–6–5	*8*	−40.17	−8.55	0.705545	0.512591	18.4323	15.6010	38.9192	0.282783
AG51–7–1	*8*	−40.17	−8.55	0.704999	0.512565	18.3014	15.5863	38.8299	
AG51–7–2	*8*	−40.17	−8.55	0.704982	0.512551	18.3086	15.5892	38.8399	0.282763
									
***Walvis Ridge Gough composition***									
CH19 DR3–2	**114**	−19.37	9.33	0.705919	0.512464	17.9161	15.5279	38.3254	0.282724
CH19 DR4–1	**113**	−19.85	9.02	0.705709	0.512342	17.5458	15.4796	38.1510	0.282668
CH19 DR4–2	**112**	−19.85	9.02	0.705607	0.512387	17.5932	15.4933	38.1276	0.282683
WALDA–002–CH19–DR4–03	*113*	−19.85	9.02	0.705574	0.512397	17.5942	15.4930	38.1328	0.282675
SO233 DR84–2	*114*	−20.35	7.64	0.706235	0.512308	17.4079	15.4574	38.0939	—
SO233 DR66–1	*107*	−22.72	7.58	0.704928	0.512612	18.2968	15.5608	38.8369	—
SO233 DR71–1	*101*	−22.67	5.13	0.704192	0.512472	18.7701	15.6325	38.9967	—
SO233 DR62–1	*96*	−25.85	6.62	0.705231	0.512519	18.1463	15.5903	38.6825	—
SO233 DR34–1–A	*87*	−26.29	3.47	0.704735	0.512585	17.9160	15.5376	38.4723	—
V29–11–1	*85*	−26.15	5.58	0.704724	0.512503	17.9811	15.5529	38.4227	0.282774
CIR 139D–2	*85*	−26.45	5.89	0.704845	0.512496	17.8002	15.5259	38.3248	0.282725
CIR 139D–3	*85*	−26.45	5.89	0.704853	0.512509	17.8275	15.5310	38.3558	0.282723
Walvis III DR04–35	*79*	−27.26	4.39	0.704678	0.512649	18.0587	15.5635	38.4937	0.282841
DSDP Leg 74 525A 53 2W 91–102	*71*	−29.07	2.99	0.704909	0.512516	17.7478	15.5081	38.2496	0.282756
DSDP Leg 74 525A 56 2W 128–134	*71*	−29.07	2.99	0.704771	0.512490	17.6759	15.4972	38.1620	0.282748
DSDP Leg 74 525A 57 5W 141–148	**72**	−29.07	2.99	0.704841	0.512489	17.6544	15.4945	38.1603	0.282747
DSDP Leg 74 525A 63 2W 59–78	*72*	−29.07	2.99	0.705061	0.512431	17.7753	15.4910	38.2252	0.282702
									
***Walvis Ridge Tristan composition***									
DSDP Leg 74 527 41 1W 47–63	*67*	−28.04	1.76	0.704144	0.512726	18.2048	15.5267	38.6715	0.282987
DSDP Leg 74 527 41 5W 95–105	*67*	−28.04	1.76	0.703608	0.512901	18.4908	15.5527	38.5336	0.283060
DSDP Leg 74 527 43 4W 59–73	*67*	−28.04	1.76	0.704547	0.512611	18.6188	15.5347	39.3297	0.282867
DSDP Leg 74 527 44 4W 59–72	*67*	−28.04	1.76	0.704427	0.512617	18.5224	15.5281	39.1353	0.282866
DSDP Leg 74 528 42 1W 29–45	*58*	−28.53	2.32	0.704744	0.512563	18.0458	15.5046	38.8273	0.282818
DSDP Leg 74 528 42 5W 31–46	**58**	−28.53	2.32	0.703996	0.512771	18.2698	15.5165	38.7332	0.283027
DSDP Leg 74 528 43 2W 80–98	**67**	−28.53	2.32	0.704092	0.512681	18.2553	15.5161	38.7148	0.282920
DSDP Leg 74 528 45 2W 109–119	*67*	−28.53	2.32	0.704285	0.512648	18.0917	15.5001	38.7408	0.282916
DSDP Leg 74 528 47 3W 66–80	*67*	−28.53	2.32	0.704456	0.512634	18.2089	15.5113	38.8174	0.282896
									
***Rio Grande rise***									
RC16–11RD 1	*84*	−30.43	−36.02	0.705578	0.512353	17.7807	15.4787	38.1734	0.282646
RC16–11RD 2	*84*	−30.43	−36.02	0.705572	0.512344	17.7866	15.4813	38.1861	0.282647
RC16–12RD 1	*84*	−30.43	−36.02	0.705687	0.512342	17.7822	15.4839	38.1913	0.282616
RC16–12RD 3	*84*	−30.43	−36.02	0.705952	0.512273	17.7127	15.4736	38.1437	0.282588
DSDP Leg 72 516F 128 2W 63–84	**85**	−30.28	−35.29	0.704988	0.512543	17.6396	15.4681	38.1425	0.282843

Full data, including errors are provided in Supplementary Dataset 1. Average 2*σ* within run errors refer to the least significant digits and are ±5 for ^87^Sr/^86^Sr, ±3 for ^143^Nd/^144^Nd, ±5 for ^176^Hf/^177^Hf, ±11, ±11 and ±33 for ^206^Pb/^204^Pb, ^207^Pb/^204^Pb and ^208^Pb/^204^Pb, respectively. External 2*σ* errors based on standards measured along with the samples are ±13 for ^87^Sr/^86^Sr, ±7 for ^143^Nd/^144^Nd, ±8 for ^176^Hf/^177^Hf, ±24, ±24 and ±61 for ^206^Pb/^204^Pb, ^207^Pb/^204^Pb and ^208^Pb/^204^Pb, respectively. ^87^Sr/^86^Sr are the least radiogenic values obtained on dual analysis of 2 N HCl leached rock chips at 70 °C for 1 h and 6 N HCl leached powders at 150° for 3 days. Ages in bold are from ref. 24. Ages in italics are either from dated samples from the same site or estimated based on a spatial linear age equation^24^.
