# Supplementary material for: How and when plume zonation appeared during the 132 Myr evolution of the Tristan Hotspot
Source: Nat Commun. 2015 Jul 27;6:7799. doi: 10.1038/ncomms8799 (PMC4525177; doi:10.1038/ncomms8799)
Supplement: Supplementary Information — Supplementary Figures 1-3 [file ncomms8799-s1.pdf]

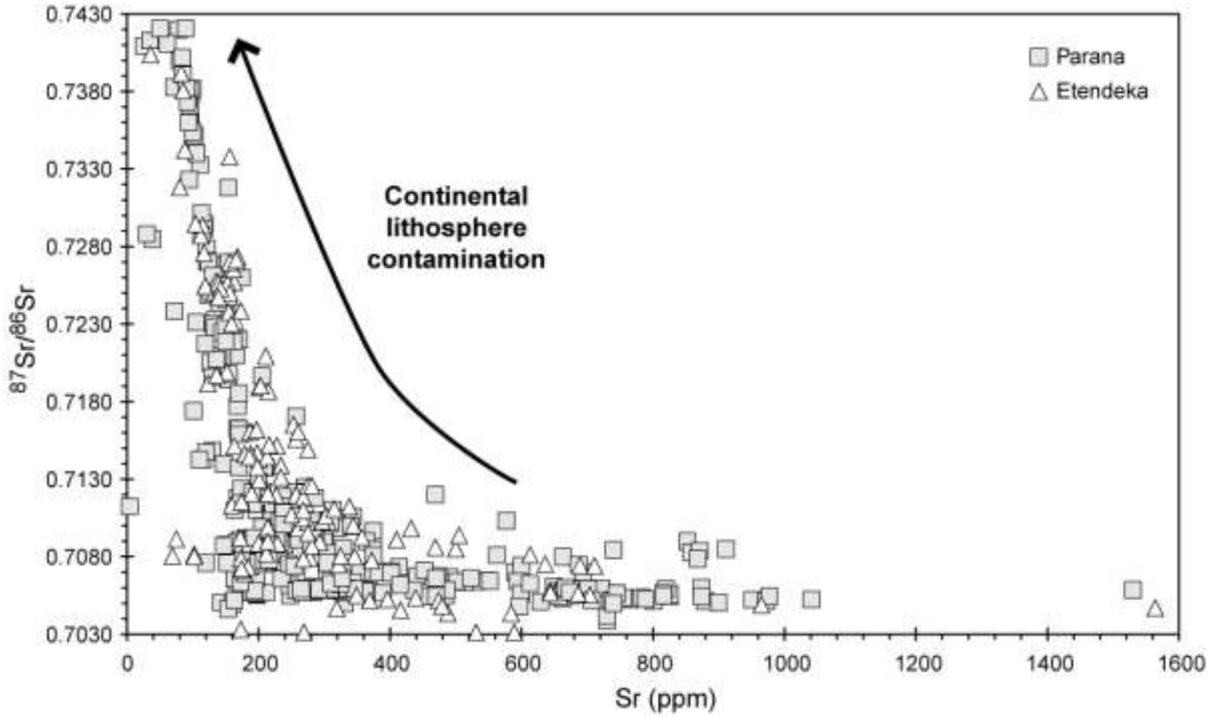

Supplementary Figure 1: Sr concentration versus $^{87}Sr/^{86}Sr$ isotope ratio for Parana and Etendeka flood basalts, showing that melts with low Sr concentrations are the most susceptible to continental lithosphere contamination.

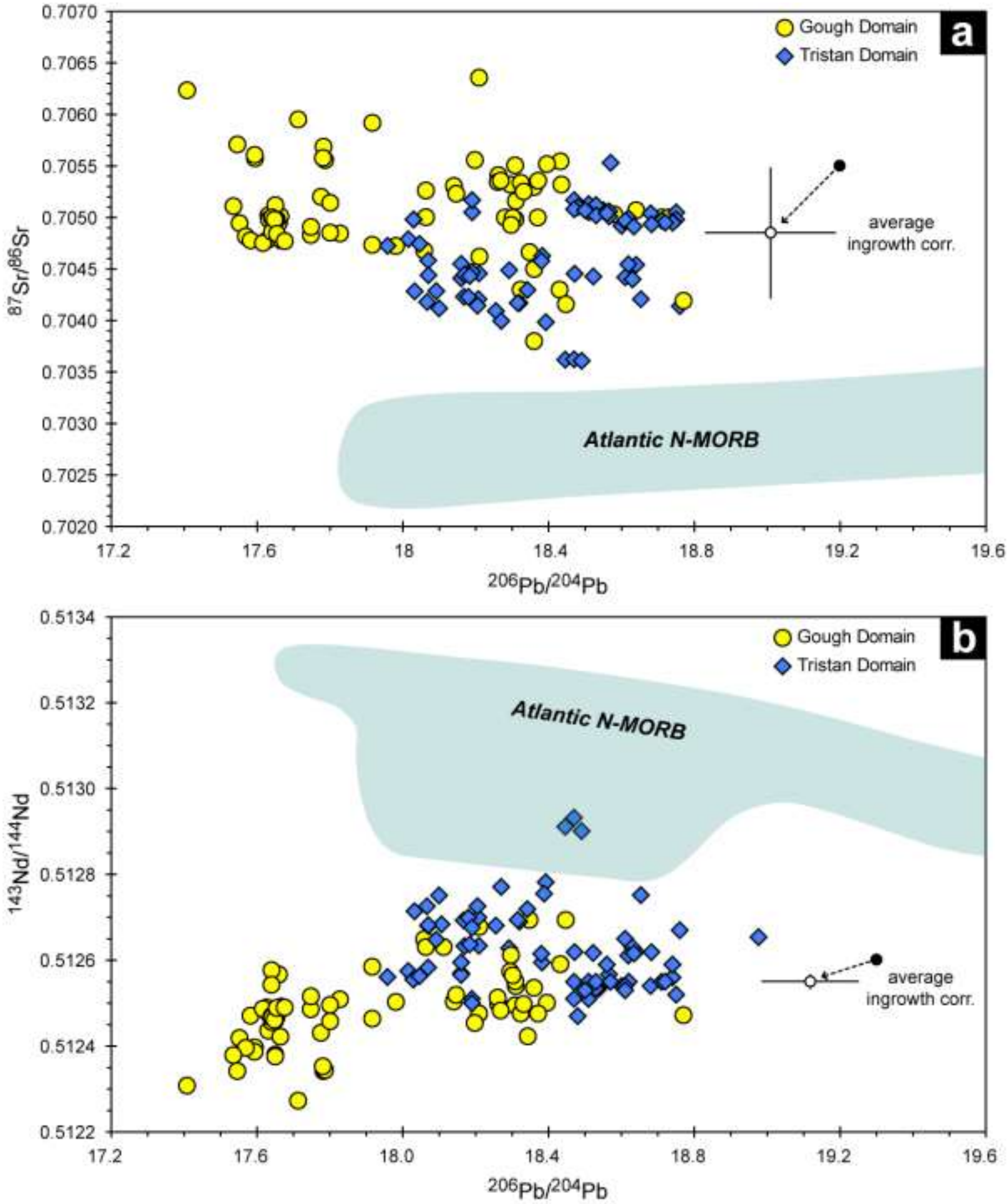

Supplementary Figure 2: Isotope correlation diagrams for **(a)** $^{206}Pb/^{204}Pb$ versus $^{87}Sr/^{86}Sr$ and **(b)** $^{206}Pb/^{204}Pb$ versus $^{143}Nd/^{144}Nd$ showing that the Tristan domain is generally shifted towards N-MORB relative to the Gough domain. Average radiogenic ingrowth correction and 1σ variation as defined in the figure 2 caption.

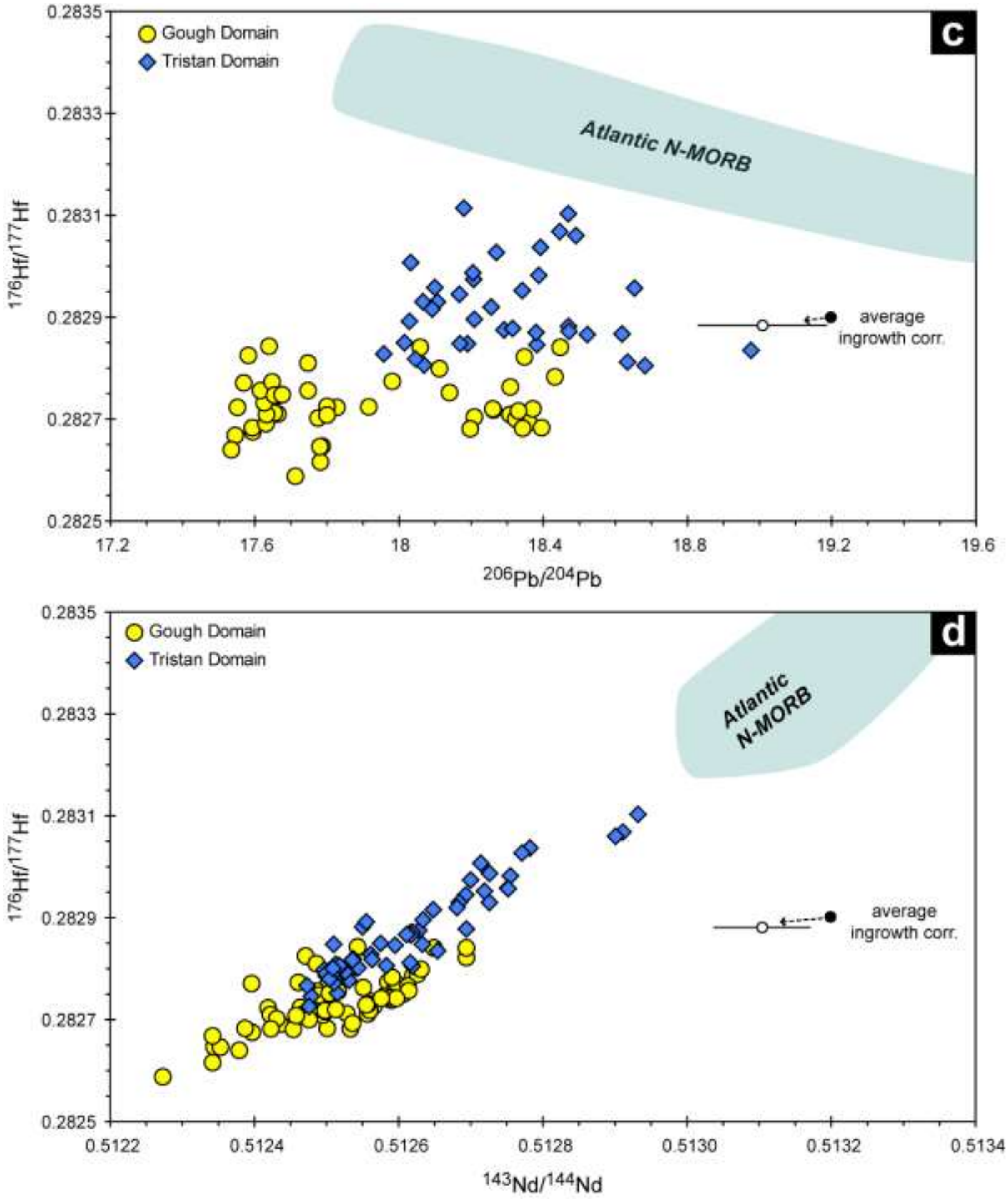

Supplementary Figure 2 continued: Isotope correlation diagrams for **(c)** $^{206}Pb/^{204}Pb$ versus $^{176}Hf/^{177}Hf$ and **(d)** $^{143}Nd/^{144}Nd$ versus $^{176}Hf/^{177}Hf$, showing that the Tristan domain is generally shifted towards N-MORB relative to the Gough domain. Average radiogenic ingrowth correction and 1σ variation as defined in the figure 2 caption.

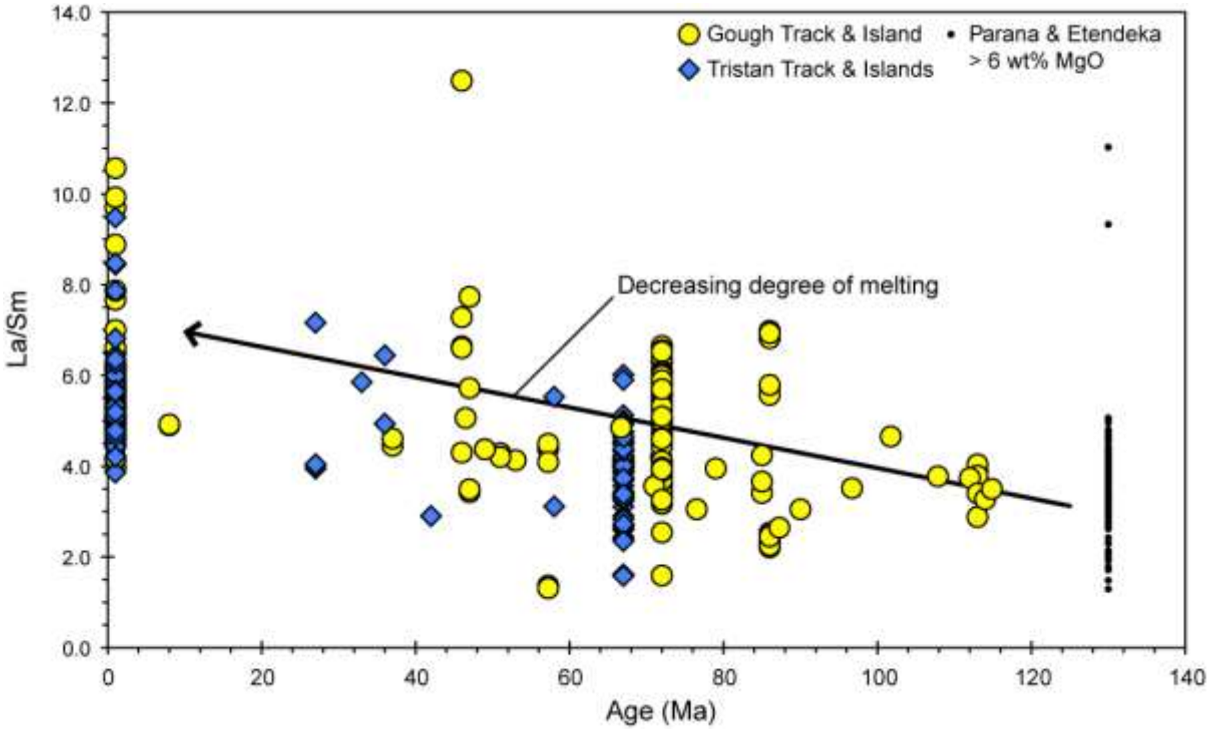

Supplementary Figure 3: Age versus La/Sm diagram for the Tristan-Gough hotspot track evolution.
